# β-Lactam Resistance Response Triggered by Inactivation of a Nonessential Penicillin-Binding Protein

**DOI:** 10.1371/journal.ppat.1000353

**Published:** 2009-03-27

**Authors:** Bartolomé Moya, Andreas Dötsch, Carlos Juan, Jesús Blázquez, Laura Zamorano, Susanne Haussler, Antonio Oliver

**Affiliations:** 1 Servicio de Microbiología and Unidad de Investigación, Hospital Son Dureta, Instituto Universitario de Investigación en Ciencias de la Salud (IUNICS) Palma de Mallorca, Spain; 2 Helmholtz Centre for Infection Research, Braunschweig, Germany; 3 Centro Nacional de Biotecnología, Consejo Superior de Investigaciones Científicas (CSIC), Campus UAM, Madrid, Spain; Massachusetts General Hospital, United States of America

## Abstract

It has long been recognized that the modification of penicillin-binding proteins (PBPs) to reduce their affinity for β-lactams is an important mechanism (target modification) by which Gram-positive cocci acquire antibiotic resistance. Among Gram-negative rods (GNR), however, this mechanism has been considered unusual, and restricted to clinically irrelevant laboratory mutants for most species. Using as a model *Pseudomonas aeruginosa*, high up on the list of pathogens causing life-threatening infections in hospitalized patients worldwide, we show that PBPs may also play a major role in β-lactam resistance in GNR, but through a totally distinct mechanism. Through a detailed genetic investigation, including whole-genome analysis approaches, we demonstrate that high-level (clinical) β-lactam resistance *in vitro*, *in vivo*, and in the clinical setting is driven by the inactivation of the *dacB*-encoded nonessential PBP4, which behaves as a trap target for β-lactams. The inactivation of this PBP is shown to determine a highly efficient and complex β-lactam resistance response, triggering overproduction of the chromosomal β-lactamase AmpC and the specific activation of the CreBC (BlrAB) two-component regulator, which in turn plays a major role in resistance. These findings are a major step forward in our understanding of β-lactam resistance biology, and, more importantly, they open up new perspectives on potential antibiotic targets for the treatment of infectious diseases.

## Author Summary

Decades after their discovery, β-lactams remain key components of our antimicrobial armamentarium for the treatment of infectious diseases. Nevertheless, resistance to these antibiotics is increasing alarmingly. There are two major bacterial strategies to develop resistance to β-lactam antibiotics: the production of enzymes that inactivate them (β-lactamases), or the modification of their targets in the cell wall (the essential penicillin-binding proteins, PBPs). Using the pathogen *Pseudomonas aeruginosa* as a model microorganism, we show that high-level (clinical) β-lactam resistance *in vitro* and *in vivo* frequently occurs through a previously unrecognized, totally distinct resistance pathway, driven by the mutational inactivation of a nonessential PBP (PBP4) that behaves as a trap target for β-lactams. We show that mutation of this PBP determines a highly efficient and complex β-lactam resistance response, triggering overproduction of the chromosomal β-lactamase AmpC and the specific activation of a two-component regulator, which in turn plays a key role in resistance. These findings are a major step forward in our understanding of β-lactam resistance biology, and, more importantly, they open up new perspectives on potential antibiotic targets for the treatment of infectious diseases.

## Introduction

Decades after their discovery, β-lactams remain key components of our antimicrobial armamentarium for the treatment of infectious diseases. Bacterial resistance to them is generally driven either by the production of enzymes that inactivate them (β-lactamases), or by the modification of their targets in the cell wall (penicillin-binding Proteins, PBPs), sometimes in conjunction with mechanisms leading to diminished permeability or active efflux [1].

While the acquisition of modified PBPs showing low affinity for β-lactams is well known to be a major resistance mechanism in Gram-positive cocci, such as penicillin-resistant *Streptococcus pneumoniae* or the much-feared methicillin-resistant *Staphylococcus aureus*, this mechanism has not been thought to be important for most species of Gram-negative rods (GNR) [2]. The production of intrinsic or horizontally acquired β-lactamases is undoubtedly the predominant resistance mechanism in the latter organisms [3]. Among GNRs, the most widely distributed β-lactamases are chromosomally-encoded AmpC variants, produced by most *Enterobacteriaceae* and *Pseudomonas aeruginosa*, high up the list of pathogens causing life-threatening infections in hospitalized patients world-wide [4].

Although AmpC is produced at very low basal levels in wild-type strains, its expression is highly inducible in the presence of certain β-lactams (β-lactamase inducers) such as cefoxitin or imipenem [3]. In fact, the efficacy of the widely-used broad spectrum penicillins (such as piperacillin) and cephalosporins (such as ceftazidime) relies on the fact that they are very weak AmpC inducers, even though they are efficiently hydrolyzed by this enzyme [3]. Unfortunately, mutants showing constitutive high level AmpC production (AmpC derepressed mutants) are frequently selected during treatment with these β-lactams, leading to the failure of antimicrobial therapy [5],[6]. In some natural strains of *Enterobacteriaceae* and *P. aeruginosa* [6]–[9], the inactivation of AmpD (cytosolic N-acetyl-anhydromuramyl-L-alanine amidase involved in peptidoglycan recycling [10]–[12]), and point mutations in AmpR (LysR-type transcriptional regulator required for *ampC* induction [13]–[15]) have been found to lead to AmpC overexpression, and thus to β-lactam resistance.

In this paper we show that, in contrast to the current expectations, the mutations triggering β-lactam resistance in *P. aeruginosa*, whether *in vitro*, *in vivo*, or in the clinical setting, frequently arise within a PBP gene. Inactivation of the *E. coli dacB* ortholog, encoding the nonessential low molecular mass PBP4 [16],[17], is demonstrated to be the principal route to one-step high level (clinical) β-lactam resistance, by triggering the overexpression of *ampC* and the specific activation of the CreBC two component regulator [18], which is also found to play a major role in resistance.

## Results/Discussion

### Mutation of *P. aeruginosa* PBP4 is the main driver of one-step high-level β-lactam resistance *in vitro* and *in vivo*

The mechanisms by which β-lactam resistance arises were studied in a previously described [19] collection of 36 independent ceftazidime resistant mutants. These mutants were obtained *in vitro* (one-step spontaneous mutants) or *in vivo* (after 3 days of treatment with humanized ceftazidime regimen in mouse model of lung infection), at two ceftazidime concentrations (4 and 16 µg/ml), and from the wild-type strain PAO1 (normal mutation rate supply) or its *mutS* deficient hypermutable derivative PAOΔ*mutS* (high mutation rate supply). In the previous study, all the mutants were shown to be highly resistant to all tested penicillins, cephalosporins, and monobactams; overexpression of the chromosomal cephalosporinase AmpC (18 to 236-fold higher expression relative to wild-type) was found to be the instrument of β-lactam resistance in all cases.

In this work, in an attempt to find out the genetic mechanisms leading to AmpC hyperproduction, we sequenced and quantified the expression of all genes so far known to be involved in *ampC* regulation and overexpression (*ampD*, *ampE*, *ampDh2*, *ampDh3* and *ampR*) in the 36 mutants. We also performed complementation experiments with plasmids harboring the wild-type *ampD* gene (pUCPAD) or the complete *ampDE* operon (pUCPADE). A complete report of the obtained results is provided in Table S1. In contrast to present models, almost none of the mutants (32 of 36) showed mutations in any of the loci examined. The only exceptions were 4 mutants obtained *in vivo* from PAOΔ*mutS* (high mutation rate supply) at the low ceftazidime concentration (4 µg/ml), each showing a different mutation in *ampD*. A modified expression of any of the studied genes was neither observed in the 32 mutants. As expected, only the four *ampD* mutants showed positive complementation with pUCPAD, but, intriguingly, all the 36 mutants showed positive complementation with pUCPADE (Table S1). Furthermore, positive complementation required the simultaneous presence of both *ampD* and *ampE*, since plasmids harboring *ampDE* operons with a non functional *ampD* and a wild-type *ampE* also failed to complement the resistance phenotype.

These findings suggested that, contrary to current understanding, mutations in *ampD* or *ampR* are not at all the most common, *in vitro* or *in vivo*, leading to AmpC overexpression and high level (clinical) β-lactam resistance in *P. aeruginosa*. Furthermore, the results strongly suggest that one-step high-level ceftazidime resistance in *P. aeruginosa* mainly occurs through single mutations in a gene/genes previously unknown to be involved in β-lactam resistance or AmpC regulation. That a single mutation has to be responsible for the resistance phenotype is shown by the ceftazidime resistance mutation rates published previously [19]. At two different ceftazidime concentrations (4 and 16 µg/ml), spontaneous resistant mutants were obtained with a rate of 10^−8^ mutations per cell division for wild-type PAO1 and of 10^−5^−10^−6^ for PAOΔ*mutS*. These mutation rates should rule out the involvement of more than one mutation in the resistance phenotype.

**Figure 1:**
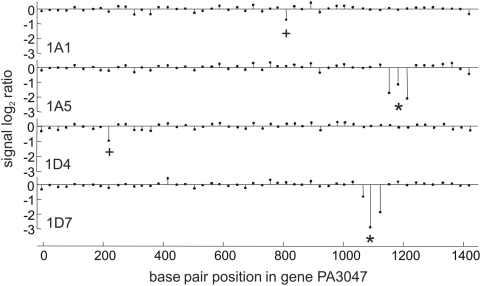
Comparative genome hybridization of four spontaneous ceftazidime-resistant mutants revealing mutations in gene PA3047. Genomic DNA from mutants 1A1, 1A5, 1D4, and 1A7 was analyzed on a whole genome DNA tiling microarray and compared to the parental wildtype PAO1. Data points (stems) represent the log_2_ ratio of signal intensity of each mutant against the wildtype signal. Mutants 1A5 and 1A7 showed strong decreases in signal at three consecutive positions (*), indicating deletions. In mutant 1A1 and 1D4, a slight decrease in signal (+) pointed towards a small genetic change, e.g., a single point mutation.

In an attempt to detect the mutations in the gene(s) yet unknown to be involved in β-lactam resistance, we followed a whole-genome analysis approach. Four of the PAO1 ceftazidime resistant *in vitro* mutants were analyzed by comparative hybridization on a recently described microarray for the discovery of single nucleotide polymorphisms (SNPs) in *P. aeruginosa* [20] using the parental PAO1 strain as reference. As shown in Figure 1, major decreases in hybridization ratios (indicating deletions of 50–100 base pairs) were detected for two of the mutants in the gene PA3047, the *E. coli dacB* ortholog, encoding the nonessential low molecular mass PBP4 [16],[17]. PCR and sequencing confirmed the presence of the deletions in this gene [(nts 1149–1231 for one of the mutants (1A5) and nts 1069–1138 for the other (1D7)] (Figure 1, Table S1). Furthermore, the two remaining mutants (1A1 and 1D4) also revealed a less pronounced decrease of hybridization ratio at a single position in gene PA3047 (Figure 1); PCR and sequencing identified as well the mutations, a G to A change in nt 819 leading to a premature stop codon (W273X) for 1A1 and a A to C change in nt 235 leading to a missense mutation (T79P) for 1D4 (Table S1).

The function and structure of PBP4 (DacB) has been characterized mainly in *E. coli*. The protein is a nonessential low molecular mass class C PBP with DD-carboxypeptidase and DD-endopeptidase activity, that is thought to play an auxiliary role in morphology maintenance, peptidoglycan maturation and recycling, and cell separation during division [17],[21],[22]. The crystal structure of *E. coli* PBP4 has been recently determined [16] and found to be organized in three domains. Domain I has the characteristic SXXK, SXN, and KTG motifs of PBPs and β-lactamases, and contains the other two extra domains embedded within it. PBP4 from *P. aeruginosa* shows a 27% identity with that of *E. coli*, and contains all conserved motifs. The alignment of *E. coli* and *P. aeruginosa* PBP4 sequences is included in Figure S1.

Following the discovery of mutations within the *dacB* ortholog, we sequenced this gene in the rest of the collection of the 36 ceftazidime resistant mutants, and the complete list of the mutations detected is provided as Table S1. All but 2 of the 32 mutants not having mutations in *ampD* had mutations in *dacB*. A total of 28 different mutations were detected, and included deletions/insertions (9), nonsense mutations (7), and missense mutations (12). Many of the missense mutations occurred in sequences encoding highly conserved motifs, including the catalytic serine, at position 72 in *P. aeruginosa* (Figure S1, Table S1).

In order to further confirm the role of *dacB* mutations in β-lactam resistance, we constructed the *dacB* knockout mutant of PAO1 (PAΔdacB). As shown in Table 1, the inactivation of *dacB* in PAO1 yielded an almost identical phenotype to that documented in the ceftazidime-resistant *dacB* spontaneous mutants, with high level β-lactam resistance and *ampC* overexpression. Therefore, it is for the first time demonstrated that the inactivation of a particular PBP (which are supposed to be antibiotic targets) produces high-level β-lactam resistance.

**Table 1:** Susceptibility to β-lactams and expression of *ampC* and *creD* genes in the studied mutants.

Strain^a^	MIC (μg/ml)^b^^,^ c						*ampC* Expression^d^^,^ e		*creD* Expression^d^^,^ e	
	CAZ	CEP	PIP	PIP/TZ	ATM	IMP	Basal	Induced	Basal	Induced
PAO1	1.5	1.5	4	2	2	1.5	1	50±14	1	36±17
PAΔC	1	1	2	2	2	0.5	NA	NA	ND	ND
PAΔR	2	2	6	4	3	0.5	3.8±0.4	3.3±0.8	1.6±0.1	189±8
PAΔD	8	4	48	32	6	2	48±4	134±11	1.1±0.1	2.7±0.7
PAΔE	1.5	1.5	4	3	2	1.5	−1.5±0.2	42±4	2.3±1.6	49±29
PAΔDE	12	4	48	32	6	2	23±8	145±43	ND	ND
PAΔDDh2Dh3	48	24	>256	>256	24	1.5	1,020±87	1,105±88	1.3±0.3	1.6±0.7
PAΔDDh2Dh3R	2	2	6	4	3	0.38	ND	ND	1.2±0.1	284±76
PAΔdacB	32	16	128	64	16	2	21±11	232±67	83±7	120±29
1A1 (DacB W273X)	32	16	128	48	16	2	58±9	211±55	53±12	112±34
1A1ΔC	0.75	0.75	1.5	1	1.5	0.38	NA	NA	ND	ND
1A1ΔR	1.5	1.5	4	3	2	0.38	1.5±0.1	1.4±0.1	47±1	103±14
1A1ΔD	128	64	>256	>256	48	2	1,770±414	1,950±480	51±24	81±40
1A1ΔE	96	64	>256	>256	32	2	296±66	665±228	58±13	78±29
2A2 (DacB frameshift)	32	16	96	64	16	2	34±16	199±54	27±12	150±29
PAΔcreBC	1.5	1.5	4	3	2	2	1.6±0.2	68±17	1.1±0.1	1.3±0.2
PAΔDDh2Dh3creBC	48	24	>256	>256	24	2	2,580±492	2,890±268	2.0±0.5	2.4±0.7
1A1ΔcreBC	4	4	24	12	4	2	40±2	210±38	1.0±0.1	1.3±0.4
2A2ΔcreBC	4	4	32	12	4	2	42±15	149±67	1.2±0.1	1.2±0.2
1A1ΔcreD	24	12	64	32	8	2	ND	ND	NA	NA

a PAO1, wild-type reference strain; PAΔC, *ampC* knockout mutant of PAO1; PAΔR, *ampR* knockout mutant of PAO1; PAΔD, *ampD* knockout mutant of PAO1; PAΔE, *ampE* knockout mutant of PAO1; PAΔDE, *ampD-ampE* knockout mutant of PAO1; PAΔDDh2Dh3, *ampD* triple (*ampD*-*ampDh2*-*ampDh3*) knockout mutant of PAO1; PAΔDDh2Dh3R, *ampR* knockout mutant of PAΔDDh2Dh3; PAΔdacB, *dacB* knockout mutant of PAO1; 1A1 (DacB W273X), DacB W273X *in vitro* spontaneous mutant of PAO1; 1A1ΔC, *ampC* knockout mutant of 1A1; 1A1ΔR, *ampR* knockout mutant of 1A1; 1A1ΔD, *ampD* knockout mutant of 1A1; 1A1ΔE, *ampE* knockout mutant of 1A1;2A2 (DacB frameshift), *in vivo* spontaneous mutant of PAO1 containing a deletion of nts 1074–1078 in *dacB*; PAΔcreBC, *creBC* knockout mutant of PAO1; PAΔDDh2Dh3creBC, *creBC* knockout mutant of PAΔDDh2Dh3creBC; 1A1ΔcreBC, *creBC* knockout mutant of 1A1; 2A2ΔcreBC, *creBC* knockout mutant of 2A2; 1A1ΔcreD, *creD* knockout mutant of 1A1.

b CAZ, ceftazidime; CEP, cefepime; PIP, piperacillin; PIP/TZ, piperacillin-tazobactam; ATM, aztreonam; IMP, imipenem.

c Clinical Laboratory Standards Institute (CLSI) susceptibility breakpoints: CAZ, CEP, and ATM≤8 µg/ml; PIP and PIP/TZ≤64 µg/ml; IMP≤4 µg/ml.

d Relative amount of *ampC* or *creD* mRNA compared to PAO1 basal level±standard deviation. Induction experiments were carried out with 50 µg/ml cefoxitin.

e NA, not applicable; ND, not determined.

### Mutation of *P. aeruginosa* PBP4 determines an AmpR-dependent overexpression of the β-lactamase AmpC

In order to understand the role of PBP4 mutation in β-lactam resistance and upregulation of AmpC expression, we constructed the *ampC*, *ampR* (transcriptional regulator of AmpC), *ampD* (negative regulator of AmpC) and *ampE* (second component of the bicistronic *ampDE* operon, encodes an inner membrane-bound sensory transducer that modulates AmpD activity [23]), knockout mutants of strain 1A1 (DacB W273X *in vitro* spontaneous mutant of PAO1) and of strain PAO1 as control. As shown in Table 1, the inactivation of *ampC* completely restored ceftazidime susceptibility in 1A1, showing that the overexpression of the β-lactamase is essential for the resistance phenotype. Furthermore, the inactivation of *ampR* restored ceftazidime susceptibility and basal *ampC* expression levels, thus demonstrating that the effect of PBP4 mutation requires a functional AmpR. Therefore, considering that PBP4 has been shown to be involved in peptidoglycan recycling [17] it seems reasonable to believe that *ampC* overexpression driven by *dacB* inactivation, as occurs in the classical *ampD* mutation pathway, should be consequence of the qualitative or quantitative modification of muropeptides, that are the effector molecules for AmpC induction through their interaction with AmpR [24]. Our results are also consistent with previous observations in the *E. coli* model, in which the strongest AmpC inducers (such as imipenem) were shown to be potent PBP4 inhibitors, suggesting a role of this PBP in the induction process [25].

Additionally, we show that the AmpDE pathway of AmpC repression is functional in the PBP4 mutants, since the inactivation of *ampD* dramatically increased further *ampC* expression and ceftazidime resistance in 1A1 (Table 1). Furthermore, while the inactivation of *ampE* in PAO1 (or in its *ampD* mutant) did not produce significant effects, it also determined a marked increase of *ampC* expression and ceftazidime resistance in the *dacB* mutant.These results suggest that both genes of the *ampDE* operon play a major role in the *dacB* mutant background. This conclusion is further supported by the positive complementation of the PBP4 mutants with the complete *ampDE* operon expressed from a multicopy plasmid (Table 2). Moreover, the expression of *dacB* from a multicopy plasmid (pUCPdB) also complemented both, the *dacB* and the *ampD* mutants (Table 2). Therefore, these results show that PBP4 and AmpDE are parallel synergic *ampC* regulatory pathways (a defect in one of them can be complemented by increasing the amount of the other), both ultimately relying on a functional AmpR.

**Table 2:** Results of the complementation experiments of the PAO1 *ampD* and *dacB* mutants with different plasmids.

Strain^a^	Ceftazidime MICs (μg/ml) When Producing the Plasmid				
	None	pUCP24 (Vector)	pUCPAD (*ampD*)	pUCPADE (*ampDE*)	pUCPdB (*dacB*)
PAO1	1.5	1.5	1.5	1.5	1.5
PAΔD	8	8	1.5	1.5	1.5
PAΔdacB	32	32	24	1.5	1.5

a PAO1, wild-type reference strain; PAΔD, *ampD* knockout mutant of PAO1; PAΔdacB, *dacB* (PBP4) knockout mutant of PAO1.

While both pathways have a very similar effect on *ampC* expression, PBP4 mutation confers high level (clinical) β-lactam resistance (i.e. resistant according to current breakpoints), while *ampD* inactivation confers only moderate resistance (i.e. still susceptible according to current resistance breakpoints) (Table 1). In fact, the resistance level conferred by PBP4 mutation is more similar to that conferred by the simultaneous inactivation of the three *ampD* genes of *P. aeruginosa* (*ampD* plus the two additional homologous genes, *ampDh2* and *ampDh3* [26]), that produces a much higher increase in *ampC* expression (Table 1). Nevertheless, this mechanism of high-level resistance is not found among clinical strains [27],[28], because it requires the acquisition of several mutations and because it causes a marked reduction of fitness and virulence [27]. Here we show that *in vivo* (murine systemic infection model) fitness is not affected in the PAO1 *dacB* mutant, as shown by the competition index (CI) of 0.92, in sharp contrast to the previously documented CIs of less than 0.01 for the double and triple *ampD* mutants [27]. Therefore, in contrast to the *ampD* inactivation pathway, PBP4 mutation in *P. aeruginosa* is a very efficient one-step trigger of high level β-lactam resistance mechanism of potentially enormous clinical relevance.

### Mutation of *P. aeruginosa* PBP4 specifically triggers the activation of the CreBC two-component regulator, which in turn plays a major role in resistance

In order to explore further the effects of PBP4 mutation, we performed a whole-genome analysis of gene expression in two selected mutant strains (1A1 and 2A2) compared to wild-type PAO1, using the Affymetrix GeneChip *P. aeruginosa* genome array. In addition to *ampC* (and co-transcribed PA4111), which obviously was upregulated, only one further gene showed a significantly (>2-fold change) modified expression. This gene, *creD*, was upregulated in both mutants analyzed (not shown). *creD* encodes an inner membrane protein of yet unknown function that is regulated by the CreBC two-component regulator. The CreBC system has been deeply studied in *E. coli*, and it is shown to be a global regulator involved in metabolic control [18]. Interestingly, the homolog of this system in *Aeromonas* Spp. (designated BlrAB) has been shown to play a role in the regulation of β-lactamase expression in these species [29]–[31]. As first approach, we analyzed whether *creD* overexpression was a signature feature of the PBP4 mutants. Indeed, real time RT-PCR experiments confirmed the overexpression of this gene in the two selected mutants (Table 1) as well as in the complete collection of *in vivo* and *in vitro* PBP4 mutants, with *creD* mRNA levels ranging from 25 to 60-fold higher than those of wild-type PAO1 (not shown). Moreover, the inactivation of *dacB* in wild-type PAO1 produced a similar *creD* overexpression (Table 1). The effect on *creD* expression seemed to be specific for the PBP4 mutants, rather than a direct consequence of AmpC overexpression, since this gene was not upregulated in the *ampD* mutants (Table 1). Interestingly, *creD* expression in wild-type PAO1 was found to be highly inducible by β-lactamase inducers (cefoxitin) (Table 1). Furthermore, *creD* inducibility was significantly reduced in the *ampD* mutants and the reduction in expression was dependent on a functional AmpR, since its inactivation restored the inducibility (Table 1). Overall, these results suggested a link between the CreBC regulator, PBP4 mutations, and the components of the regulatory system of *ampC* expression.

The complete *creB*, *creC* and *creD* genes, as well as their promoter regions, were fully sequenced in 1A1, 2A2 and five additional randomly selected mutants from the collection. The absence of mutations supported further the notion that the mutations in PBP4 are solely responsible for the complete β-lactam resistance response. The single mutation hypothesis is definitively confirmed by the fact that direct *dacB* inactivation produces the same phenotype (i.e. the same MICs and *ampC* and *creD* expression levels) observed in the spontaneous *dacB* mutants.

To gain insights into the role of the CreBC system in β-lactam resistance, we constructed *creBC* and *creD* knockout mutants of the PBP4 mutants 1A1 and 2A2, as well as of PAO1 and its single and triple *ampD* mutants as controls. Interestingly, the inactivation of *creBC* in the PBP4 mutants (1A1 and 2A2) not only decreased *creD* expression back to wild-type levels, but also drastically decreased β-lactam MICs, leaving them well within the susceptible (treatable) range according to current breakpoints (Table 1). Furthermore, the effect was specific to the PBP4 mutants, since β-lactam susceptibility was not affected by *creBC* inactivation in wild-type PAO1 or in its *ampD* mutants (Table 1). Overall, these results strongly suggest that PBP4 mutations specifically trigger the activation of the CreBC two-component regulator, leading to *creD* upregulation and β-lactam resistance. Nevertheless, CreD seems not to be the only CreBC-dependent driver of resistance, since the direct inactivation of *creD* in the PBP4 mutants decreased resistance slightly, but did not give the drastic reduction seen on CreBC inactivation (Table 1). The extra resistance of the PBP4 mutants compared to the *ampD* mutant (despite showing similar levels of *ampC* expression) is therefore apparently driven by the specific activation of the CreBC system in the PBP4 mutants. Indeed, the resistance level of 1A1 after CreBC inactivation was more similar to that of the *ampD* mutant (Table 1). Further evidence showing that the CreBC system is a key component in one-step high level β-lactam resistance development was provided by mutation rates experiments. While high level ceftazidime resistant mutants were readily selected from PAO1 wild-type strain [mutation rate to ceftazidime (at breakpoint concentration, 16 µg/ml) resistance of 3×10^−8^ mutants per cell division], mutation rates were below the detection limit (<1×10^−11^) for its CreBC knockout mutant (PAΔCreBC), consistently with the interpretation that a functional CreBC system is required for one-step high level (clinical) β-lactam resistance development in *P. aeruginosa*.

Nevertheless, in contrast to the previous experiences with the BlrAB system in *Aeromonas* Spp., CreBC mediated resistance was not directly driven by an effect on *ampC* expression, since 1A1ΔCreBC (despite showing a drastic reduction of resistance) had similar (still overexpressed) *ampC* levels than the parent 1A1 (Table 1). Moreover, β-lactamase activity was also similar (data not shown), showing that apparently the effect is neither produced by posttranscriptional modification of AmpC. Therefore, although we demonstrate that mutation of PBP4 specifically activates the CreBC two-component regulator, and that this event plays a major role in β-lactam resistance, the underlying mechanism is still uncertain. Despite only *creD* showed a modified expression greater 2-fold in the transcriptome analysis, even small modifications of expression of genes involved in outer membrane permeability, antibiotic efflux or general metabolism could significantly enhance the effect of AmpC overexpression and thus β-lactam resistance. In any case, our findings indicate that the nomenclature for this two-component system in *P. aeruginosa* should be changed to follow that used for *Aeromonas* Spp. (Blr, standing for β-lactam resistance) and not that used in *E. coli* (Cre, standing for carbon-source responsive) [18], [29]–[31].

**Figure 2:**
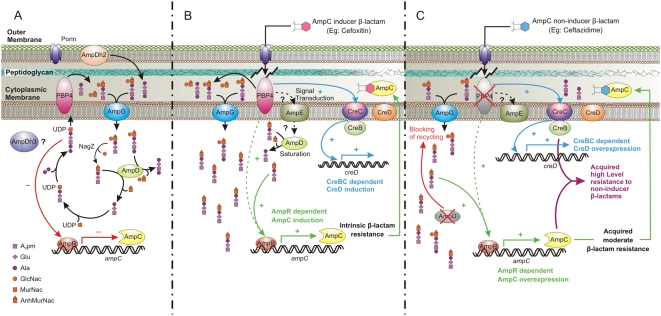
Schematic representation of the regulation of the *P. aeruginosa* chromosomal β-lactamase AmpC and peptidoglycan recycling under different conditions. (A) Wild-type strain in the absence of β-lactams. During regular bacterial growth, the peptidoglycan degradation products, MurNac-peptides [N-acetylglucosaminyl-1,6-anhydro- N-acetylmuramyl-tri (or tetra) peptides], are generated in the periplasm through the activity of PBP4 and several other enzymes. These products are then internalized through the permease AmpG, and processed in the cytoplasm by the β-N-acetylglucosaminidase NagZ and the N-acetyl-anhydromuramyl-L-alanine amidase AmpD. *P. aeruginosa* has two additional AmpD proteins, AmpDh2 that it is apparently located in the outer membrane and AmpDh3 that is still of unknown location. The generated tripeptides are then incorporated to the murein biosynthesis pathway to yield the UDP-MurNac-pentapeptides that will be exported to the periplasm and incorporated to the peptidoglycan, to complete the recycling process. In the absence of β-lactam antibiotics, these UDP-MurNac-pentapeptides interact with AmpR, which functions as a negative regulator of *ampC* expression. (B) Growth of wild-type strain in the presence AmpC inducer β-lactams. During growth in the presence of certain β-lactams (AmpC inducers), such as cefoxitin or imipenem, AmpC is produced at high levels, conferring natural (intrinsic) resistance to the antibiotic, provided it is a good substrate for the enzyme (as occurs for cefoxitin but not for imipenem). The exact mechanism responsible for the induction of the expression of AmpC in the presence of these antibiotics is still not fully understood. One of the components of the induction process is apparently the saturation of AmpD, due to the enhanced generation of its substrate (MurNac-tripeptides). The accumulated MurNAc-tripeptides are thought to displace the UDP-MurNAc-pentapeptides from AmpR, converting it into an activator of *ampC* transcription. Our results, and other previous indirect evidences, suggest that the inhibition of PBP4 by these β-lactam antibiotics (known to be the most potent PBP4 inhibitors) plays a major role in the *ampC* induction process, and determines the activation of the CreBC (BlrAB) two-component regulator. The exact function of the signal transducer AmpE, located in the inner membrane, still needs to be elucidated, but apparently interacts with both AmpD and PBP4. (C) Growth of the AmpD and/or PBP4 mutants in the presence of AmpC non-inducer β-lactams (most antipseudomonal cephalosporins and penicillins, such as ceftazidime or piperacillin, respectively). The inactivation of PBP4 or AmpD produces a very similar constitutive *ampC* overexpression. Both mechanisms ultimately relay in the activation of AmpR, which changes its activity from negative to positive regulator of *ampC* expression. Independently of the mechanism, AmpC overexpression itself is shown to confer only moderate (low-level) acquired resistance to non-inducer β-lactams. Additionally, the inactivation of PBP4 specifically activates the CreBC (BlrAB) system, which drives, in conjunction with the AmpC overexpression, the high-level β-lactam resistance response.

Figure 2 summarizes the current knowledge on *P. aeruginosa ampC* regulation, peptidoglycan recycling, and the described similarities and differences of the β-lactam resistance response driven by AmpD inactivation or PBP4 mutation.

### Role of PBP4 mutation in the clinical setting

To find out whether PBP4 mutations and the linked CreBCD mediated resistance documented for *in vitro* and *in vivo* mutants occurred also in natural human infections, we investigated a previously described collection of clinical strains [6]. This collection included 10 isogenic pairs of ceftazidime susceptible and resistant clinical isolates obtained from 10 Intensive Care Unit patients. All patients had severe *P. aeruginosa* infections that were treated with β-lactams, experiencing therapy failure due to resistance development. In all cases, the subsequent ceftazidime resistant isolate showed AmpC hyperproduction, but only in four of them could the resistance phenotype be attributed to known mechanisms (*ampD* mutations) [6]. As shown in Table 3, all 6 remaining ceftazidime isolates contained mutations in *dacB* (not present in the preceding isogenic susceptible isolate). Interestingly, two of the isolates (despite them being genetically distinct) have sustained the same PBP4 mutation (T428P); this same mutation was found in one of the PAO1 *in vivo* mutants (Table S1) and involves a conserved residue close to the KTG motif [16]. Furthermore, consistent with the findings for *in vitro* and *in vivo* mutants, all six natural PBP4 mutants overexpressed *creD* (2.8–38 fold higher expression than their respective wild-type isolates) (Table 3). The inactivation of *creBC* significantly reduced ceftazidime resistance in all but one of the natural PBP4 mutants (Table 3). On the other hand, the expression of *creD* was not modified in the four clinical strains containing only mutations in *ampD* (not shown).

**Table 3:** Characterization of β-lactam resistant clinical isolates.

Isogenic CAZ S/CAZ R Sequential Isolates^a^^,^ b	β-Lactam Treatment before Resistance^b^^,^ c	Fold Increase β-Lactamase Activity^c^^,^ d	Mutations [Effect]^c^^,^ e *ampD*	Mutations [Effect]^c^^,^ e *dacB*	CAZ MIC (µg/ml) in CAZ S/CAZ R/CAZ R (pUCPdB)/CAZ R ΔCreBC^f^				*creD* Expression in CAZ S/CAZ R	
MCV1C4/MCV1C6	PIP-TZ	26.7	None	Del. nts 1077–1081 [Deletion/frameshift]	1	24	2	8	1.2±0.1	14.6±4.0^h^
FMC1H1/FMC1H6	CAZ, PIP-TZ	137.3	None	T-C nt −79 [mutation in putative regulatory region]	1	16	1.5	Not obtained g	1.0±1.8	21.8±7.6^h^
VFF2D5/VFF2E2	CAZ	13.0	None	G-A nt 275 [W92X]	1.5	32	1.5	1.5	1.0±1.6	38.1±10.7^h^
MSF2F4/MSF2F5	IMP, CAZ	11.7	None	A-C nt 1282 [T428P]	1.5	16	2	8	1.1±0.1	3.2±0.9^h^
JSG1H9/JSG2A1	IMP, PIP-TZ	516.0	Ins. 1-bp (C) in nt 481 [frameshift]	A-C nt 1282 [T428P]	6	>256	48	>256	1.2±0.3	17.4±8.4^h^
OFC2H1/OFC2I4	CAZ, CEP	304.0	Complete deletion of *ampDE*	Del. nts 600–6002 [M200I, Del. D2001]	4	96	24	32	1.0±1.5	3.2±0.4^h^

a CAZ, ceftazidime; PIP-TZ, piperacillin-tazobactam; IMP, imipenem; CEP, cefepime.

b Strains JSG1H9/JSG2A1 and OFC2H1/OFC2I4 additionally overexpressed the efflux pumps MexAB-OprM and MexEF-OprN, respectively [6].

c The information on antibiotic treatments, β-lactamase activity, and *ampD* mutations was obtained from previous work [6].

d Fold increase of beta-lactamase activity in the ceftazidime-resistant isolate compared to its preceding clonally related susceptible isolate.

e Mutations detected in the ceftazidime-resistant isolates not present in the preceding isogenic susceptible isolates. None of the isolates contained mutations in *ampR* or *ampC-ampR* intergenic region.

f Ceftazidime MIC (µg/ml) of: ceftazidime-susceptible isolates, subsequent ceftazidime-resistant clonally related isolates, ceftazidime-resistant isolates complemented with plasmid pUCPdB harbouring the wild-type *dacB* gene, and CreBC knockout mutants of the ceftazidime-resistant isolates.

g CreBC knockout mutants of this strain were not obtained after several attempts.

h p<0.05 for the comparison of *creD* expression in CAZ R – CAZ S pairs of sequential isolates.

### Concluding remarks

Using *P. aeruginosa* as a model organism, we have shown that the most prevalent mutations causing immediate onset of high level β-lactam resistance are found in the *dacB* gene, encoding the nonessential PBP4. This is the first demonstration of the acquisition of β-lactam resistance through such a mechanism. All the previous examples by which PBP-mediated resistance develops involve modified enzymes showing low affinity for β-lactams (target modification) [2]. Even though inactivation of the classical AmpC negative regulator AmpD upregulates AmpC, only mutations in *dacB* confer high level (clinical) β-lactam resistance. The inactivation of PBP4 is found to trigger an AmpR-dependent overproduction of the chromosomal β-lactamase AmpC, and the specific activation of the CreBC (BlrAB) two-component regulator, which in turn plays a major role in the β-lactam resistance response. This interplay between mutation of supposed antibiotic targets, production of antibiotic inactivating enzymes, and global regulators, is an unexpected layer of complexity of β-lactam resistance biology, which provides new perspectives on potential antibiotic targets for the treatment of infectious diseases. Since all the components of the described resistance mechanism (*dacB*, *ampC*, *ampR*, and *creBCD*) are found in the genomes of many GNR (*E. coli* might be an example of exception since *ampR* is not present in this species [14]), the results presented here are expected to have broad implications for the development of new antimicrobial compounds. Particularly, the CreBCD system is envisaged as an attractive candidate target to develop molecules capable of reducing the development of resistance when used together with β-lactam antibiotics.

## Materials and Methods

### Bacterial strains and plasmids

A complete list of laboratory strains and plasmids used or constructed in this study is provided as Table S2. A previously described [19] collection of 36 independent ceftazidime resistant mutants was used. These mutants were obtained either *in vitro* (one-step spontaneous mutants) or *in vivo* (after 3 days of treatment with humanized ceftazidime regimen in mouse model of lung infection), at two ceftazidime concentrations (4 and 16 µg/ml), and from the wild-type strain PAO1 (normal mutation rate supply) or its *mutS* deficient derivative PAOΔ*mutS* (high mutation rate supply). Additionally, a previously reported [6] collection of 10 isogenic pairs of ceftazidime susceptible and resistant clinical isolates obtained from 10 Intensive Care Unit patients suffering from severe *P. aeruginosa* infections was used.

### Construction of knockout mutants

*P. aeruginosa* single or multiple knockout mutants in *ampD*, *ampE*, *ampR*, *ampC*, *creBC*, *creD*, or *dacB* were constructed using the *Cre-lox* system for gene deletion and antibiotic resistance marker recycling [32]. Upstream and downstream PCR products (primers used provided as Table S3) of each gene were digested with either BamHI or EcoRI and HindIII and cloned by a three way ligation into pEX100Tlink deleted for the HindIII site and opened by EcoRI and BamHI. The resulting plasmids were transformed into *E. coli* XL_1_Blue strain and transformants were selected in 30 µg/ml ampicillin LB agar plates. The *lox* flanked gentamicin resistance cassette (*aac1*) obtained by HindIII restriction of plasmid pUCGm*lox* was cloned into the single site for this enzyme formed by the ligation of the two flanking fragments. The resulting plasmids were again transformed into *E. coli* XL_1_Blue strain and transformants were selected in 30 µg/ml ampicillin-5 µg/ml gentamicin LB agar plates. Plasmids were then transformed into the *E. coli* S17-1 helper strain. Knockout mutants were generated by conjugation followed by selection of double recombinants using 5% sucrose-1 µg/ml cefotaxime-30 µg/ml gentamicin LB agar plates. Double recombinants were checked first screening for carbellicin (200 µg/ml) susceptibility and afterwards by PCR amplification and sequencing. For the recycling of the gentamicin resistance cassettes, plasmid pCM157 was electroporated into the different mutants. Transformants were selected in 250 µg/ml tetracycline LB agar plates. One transformant for each mutant was grown overnight in 250 µg/ml tetracycline LB broth in order to allow the expression of the *cre* recombinase. Plasmid pCM157 was then cured from the strains by successive passages on LB broth. Selected colonies were then screened for tetracycline (250 µg/ml) and gentamicin (30 µg/ml) susceptibility and checked by PCR amplification and DNA sequencing.

### PCR, sequencing, and quantification of gene expression

In order to explore the β-lactam resistance mechanisms in the above described collection of bacterial strains, *ampD*, *ampE*, *ampR*, *ampDh2*, *ampDh3*, *creB*, *creC*, *creD* and *dacB* genes were amplified by PCR, using primers described in Table S3, and fully sequenced. All mutations detected were checked by sequencing a fresh independent PCR product. Sequencing reactions were performed with the BigDye Terminator Kit (PE Applied Biosystems, Foster City, Calif.) and sequences were analyzed on an ABI prism 3100 DNA sequencer (PE Applied Biosystems). Resulting sequences were then compared (www.ncbi.nih.gov/BLAST) with those of the wild-type PAO1 strain [33],[34].

The levels of expression of *ampC*, *ampD*, *ampE*, *ampDh2*, *ampDh3* and *creD* were determined by real time RT-PCR with and without cefoxitin induction. Total RNA from logarithmic-phase-grown cultures (grown with and without 50 µg/ml cefoxitin) was obtained with the RNeasy Mini Kit (QIAGEN, Hilden, Germany) and treated with 2 U of TURBO DNase (Ambion) for 30 min at 37°C to remove contaminating DNA. The reaction was stopped by the addition of 5 µl of DNase inactivation reagent and the samples were adjusted to a final concentration of 50 ng/µl. A 500 ng sample of purified RNA was then used for one-step reverse transcription and real-time PCR amplification using the QuantiTect SYBR Green RT-PCR Kit (QIAGEN, Hilden, Germany) in a SmartCycler II (Cepheid, Sunnyvale, CA). The primers listed in Table S3 were used for amplification of *ampC*, *ampD*, *ampE*, *ampDh2*, *ampDh3*, *creD*, and *rpsL* (used as reference to normalize the relative amount of mRNA). In all cases, the mean values of relative mRNA expression obtained in at least three independent duplicate experiments were considered.

### Susceptibility testing, quantification of β-lactamase activity, complementation studies, estimation of mutation rates, and fitness experiments

Minimal inhibitory concentrations (MICs) for ceftazidime, cefepime, ticarcillin, piperacillin, piperacillin/tazobactam, aztreonam, imipenem, meropenem ciprofloxacin, tobramycin, tetracycline, chloramphenicol and colistin were determined in Müller-Hinton (MH) agar plates using E-test strips (AB Biodisk, Sweden) following the manufacturers recommendations. β-lactamase specific activity (nanomoles of nitrocefin hydrolyzed per minute per milligram of protein) was determined spectrophotometrically on crude sonic extracts as previously described [6]. To determine the β-lactamase specific activity after induction, before the preparation of the crude sonic extracts, the strains were grown in the presence of 50 µg/ml cefoxitin for 3 h. In all cases, the mean β-lactamase activity values obtained in three independent experiments were considered. Complementation experiments were performed following previously described protocols [6]. Briefly, plasmids pUCPAD (harboring the wild-type *ampD* gene), pUCPADE (harboring the complete wild-type *ampDE* operon) or pUCP24 (cloning vector) were electroporated into the different β-lactam resistant strains or PAO1 (as control). Additionally, plasmids pUCPADE2A1, pUCPADE1C5 or pUCPADE2C2 containing a non functional *ampD* and a wild-type *ampE* were electroporated in selected mutants. Finally, complementation experiments using the cloned wild-type *dacB* gene (pUCPdB) were also performed in selected strains. Transformants were selected in 50 µg/ml gentamicin LB agar plates. Complementation was considered positive when the MICs of ceftazidime for the transformants were at least 3 two-fold dilutions lower than those of the parent strains. The rates of mutation to high level (16 µg/ml) ceftazidime resistance were estimated following previously described protocols (19). To determine the effect on bacterial fitness of particular resistance mutations, *in vitro* (LB growth) and *in vivo* (murine model of systemic infection) competition experiments were performed following previously described procedures [27]. Median Competition Indexes (CIs), defined as the mutant/wild-type ratio, were calculated from at least 8 independent experiments.

### Analysis of whole-genome gene expression

Three independent replicates of PAO1 and of two selected ceftazidime resistant mutants (1A1 and 2A2) were grown in 10 ml of LB broth in a 50-ml baffled flask vigorously shaken at 37°C to an optical density at 600 nm (OD600) of 1. The cells were collected by centrifugation (8,000 g for 5 min at 4°C) and total RNA was isolated using the RNeasy minikit (QIAGEN) following the manufacturer's instructions. RNA was dissolved in water and treated with 2 U of TURBO DNase (Ambion) for 30 min at 37°C to remove contaminating DNA. The reaction was stopped by the addition of 5 µl of DNase inactivation reagent. Ten micrograms of total RNA were checked by running on an agarose gel prior to cDNA synthesis. cDNA synthesis, fragmentation, labeling and hybridization were performed according to the Affymetrix GeneChip *P. aeruginosa* genome array expression analysis protocol. Expression analysis was performed as described previously [35]. Only transcripts showing higher than two-fold increases or decreases were considered as differentially expressed. In all cases the PPDE (posterior probability for differential expression) was between 0.999 and 1.

### Whole-genome analysis to detect the mutations involved in ceftazidime resistance

In order to detect the presence of mutations in genes yet unknown to be involved in β-lactam resistance, a whole-genome analysis approach was followed. For this purpose, four ceftazidime resistant mutants were analyzed and compared with wild-type PAO1 using a recently described microarray for the discovery of single nucleotide polymorphisms (SNPs) in *P. aeruginosa* [20]. Cultures were grown in brain-heart infusion (BHI) medium for 12 h at 37°C in shaking glass flasks at 180 rpm and genomic DNA was isolated using the DNeasy Blood & Tissue Kit (Qiagen). Cell lysates were treated with RNase I (Qiagen) to prevent accidental carryover of RNA to the microarray. Genomic DNA was partially digested with DNase I (Amersham Biosciences, Piscataway, NJ) to a fragment size of ∼50–250 bp, confirmed by gel electrophoresis, and fragments were labeled at the 3′-ends with biotin-ddUTP (Roche Diagnostics, Indianapolis, IN) using Terminal deoxynucleotidyl transferase (Roche). Samples were hybridized to an identical lot of PATA1 arrays [20] for 16 hours at 50°C. After hybridization the microarrays were washed, stained with SA-PE and read using an Affymetrix GeneChip fluidic station and scanner according to Affymetrix standard protocols (Affymetrix, Santa Clara, CA). Analysis of microarray data was performed using the Affymetrix GCOS 1.4 to generate the raw data files (cel data). The raw data files were further analyzed using ‘Tiling Analysis Software’ (TAS) version 1.1 by Affymetrix.

## Supporting Information

Table S1 Characterization of *in vitro* and *in vivo* ceftazidime-resistant mutants. The sequences corresponding to the signal peptides are shown in italics. SXXK, SXN, and KTG motifs are shown within boxes and the delimitations of the three domains are indicated with arrows. Asterisks, colons, and periods indicate identical, conserved, and semiconserved residues, respectively.
(0.06 MB PDF)

Table S2 Strains and plasmids used or constructed in this study
(0.06 MB PDF)

Table S3 Primers used in this work
(0.04 MB PDF)

Figure S1 Clustal W 2.0.8 multiple-sequence alignment of DacB from *E. coli* K12 and *P. aeruginosa* PAO1
(0.03 MB PDF)

## References

[1] Poole K (2004). Resistance to β-lactam antibiotics.. Cell Mol Life Sci.

[2] Zapun A, Contreras-Martel C, Vernet T (2008). Penicillin-binding proteins and β-lactam resistance.. FEMS Microbiol Rev.

[3] Livermore DM (1995). β-lactamases in laboratory and clinical resistance.. Clin Microbiol Rev.

[4] Vincent JL (2003). Nosocomial infections in adult intensive-care units.. Lancet.

[5] Livermore DM (1987). Clinical significance of beta-lactamase induction and stable derepression in gram-negative rods.. Eur J Clin Microbiol.

[6] Juan C, Macia MD, Gutierrez O, Vidal C, Perez JL (2005). Molecular mechanisms of β-lactam resistance mediated by AmpC hyperproduction in *Pseudomonas aeruginosa* clinical strains.. Antimicrob Agents Chemother.

[7] Stapleton P, Shannon K, Phillips I (1995). DNA sequence differences of *ampD* mutants of *Citrobacter freundii*.. Antimicrob Agents Chemother.

[8] Bagge N, Ciofu O, Hentzer M, Campbell JI, Givskov M (2002). Constitutive high expression of chromosomal β-lactamase in *Pseudomonas aeruginosa* caused by a new insertion sequence (IS*1669*) located in *ampD*.. Antimicrob Agents Chemother.

[9] Langaee TY, Cagnon L, Huletsky A (2000). Inactivation of the *ampD* gene in *Pseudomonas aeruginosa* leads to moderate-basal-level and hyperinducible AmpC β-lactamase expression.. Antimicrob Agents Chemother.

[10] Lindberg F, Lindquist S, Normark S (1987). Inactivation of the *ampD* gene causes semiconstitutive overproduction of the inducible *Citrobacter freundii* β-lactamase.. J Bacteriol.

[11] Höltje JV, Kopp U, Ursinus A, Wiedemann B (1994). The negative regulator of β-lactamase induction AmpD is a N-acetyl-anhydromuramyl-L-alanine amidase.. FEMS Microbiol Lett.

[12] Normark S (1995). β-lactamase induction in Gram-negative bacteria is intimately linked to peptidoglycan recicling.. Microb Drug Resist.

[13] Lindberg F, Westman L, Normark S (1985). Regulatory components in *Citrobacter freundii ampC* β-lactamase induction.. Proc Natl Acad Sci USA.

[14] Honore N, Nicolas MH, Cole ST (1986). Inducible cephalosporinase production in clinical isolates of *Enterobacter cloacae* is controlled by a regulatory gene that has been deleted from *Escherichia coli*.. EMBO J.

[15] Kong KF, Jayawardena SR, Indulkar SD, del Puerto A, Koch CL (2005). *Pseudomonas aeruginosa* AmpR is a global transcriptional factor that regulates expression of AmpC and PoxB beta-lactamases, proteases, quorum sensing, and other virulence factors.. Antimicrob Agents Chemother.

[16] Kishida H, Unzai S, Roper DI, Lloyd A, Park SY (2005). Crystal structure of penicillin binding protein 4 (dacB) from *Escherichia coli*, both in the native form and covalently linked to various antibiotics.. Biochemestry.

[17] Ghosh AS, Chowdhury C, Nelson DE (2008). Physiological functions of D-alanine carboxipeptidases in *Escherichia coli*.. Trends Microbiol.

[18] Avison MB, Horton RE, Walsh TR, Bennett PM (2001). *Escherichia coli* CreBC is a global regulator of gene expression that responds to growth in minimal media.. J Biol Chem.

[19] Plasencia V, Borrell N, Maciá MD, Moya B, Pérez JL (2007). Influence of high mutation rates on the mechanisms and dynamics of in vitro and in vivo resistance development to single or combined antipseudomonal agents.. Antimicrob Agents Chemother.

[20] Dötsch A, Pommerenke C, Bredenbruch F, Geffers R, Häussler S (2009). Evaluation of a microarray-hybridyzation based method applicable for discovery of single nucleotide polymorphisms (SNPs) in the *Pseudomonas aeruginosa* genome.. BMC genomics.

[21] Korat B, Mottl M, Kech W (1991). Penicillin-binding protein 4 of *Escherichia coli*: molecular cloning of the *dacB* gene, controlled overexpression, and alterations in murein composition.. Mol Microbiol.

[22] Meberg BM, Paulson AL, Privadarshini R, Young KD (2004). Endopeptidase penicillin-binding proteins 4 and 7 play auxiliary roles in determining uniform morphology of *Escherichia coli*.. J Bacteriol.

[23] Honore N, Nicolas MH, Cole ST (1996). Regulation of enterobacterial cephalosporinase production: the role of a membrane-bound sensory transducer.. Mol Microbiol.

[24] Jacobs C, Huang LJ, Bartowsky E, Normark S, Park JT (1994). Bacterial cell wall recycling provides cytosolic muropeptides as effectors for beta-lactamase induction.. EMBO J.

[25] Sanders CC, Bradford PA, Ehrhardt AF, Bush K, Young KD (1997). Penicillin-binding proteins and induction of AmpC beta-lactamase.. Antimicrob Agents Chemother.

[26] Juan C, Moya B, Perez JL, Oliver A (2006). Stepwise upregulation of the *Pseudomonas aeruginosa* chromosomal cephalosporinase conferring high level beta-lactam resistance involves three AmpD homologues.. Antimicrob Agents Chemother.

[27] Moya B, Juan C, Alberti S, Perez JL, Oliver A (2008). Benefit of having multiple *ampD* genes for acquiring β-lactam resistance without losing fitness and virulence in *Pseudomonas aeruginosa*.. Antimicrob Agents Chemother.

[28] Schmidtke AJ, Hanson ND (2008). Role of ampD homologs in overproduction of AmpC in clinical isolates of *Pseudomonas aeruginosa*.. Antimicrob Agents Chemother.

[29] Niumsup P, Simm AM, Nurmahomed K, Walsh TR, Bennett PM (2003). Genetic linkage of the penicillinase gene, *amp*, and *blrAB*, encoding the regulador of β-lactamase expression in *Aeromonas* Spp.. J Antimicrob Chemother.

[30] Avison MB, Niumpsup P, Nurmahomed K, Walsh TR, Bennett PM (2004). Role of the ‘cre/blr-tag’ DNA sequence in regulation of gene expression by the *Aeromonas hydrophila* beta-lactamase regulator, BlrA.. J Antimicrob Chemother.

[31] Alksne LE, Rasmussen BA (1996). Expression of the AsbA1, OXA-12, and AsbM1 beta-lactamases in *Aeromonas jandaei* AER 14 is coordinated by a two-component regulon.. J Bacteriol.

[32] Quénée L, Lamotte D, Polack B (2005). Combined *sacB*-based negative selection and cre-lox antibiotic marker recycling for efficient gene deletion in *Pseudomonas aeruginosa*.. BioTechniques.

[33] Stover CK, Pham XQ, Erwin AL, Mizoguchi SD, Warrener P (2000). Complete genome sequence of *Pseudomonas aeruginosa* PAO1: an opportunistic pathogen.. Nature.

[34] Winsor GL, Lo R, Sui SJ, Ung KS, Huang S (2005). *Pseudomonas aeruginosa* genome database and PseudoCAP: facilitating community-based, continually updated, genome annotation.. Nucleic Acids Res.

[35] Blazquez J, Gómez-Gómez JM, Oliver A, Juan C (2006). PBP3 inhibition elicits adaptive responses in *Pseudomonas aeruginosa*.. Mol Microbiol.

