# Supplementary material for: β-Lactam Resistance Response Triggered by Inactivation of a Nonessential Penicillin-Binding Protein
Source: PLoS Pathog. 2009 Mar 27;5(3):e1000353. doi: 10.1371/journal.ppat.1000353 (PMC2654508; doi:10.1371/journal.ppat.1000353)
Supplement: Table S1 — Characterization of in vitro and in vivo ceftazidime-resistant mutants. The sequences corresponding to the signal peptides are shown in italics. SXXK, SXN, and KTG motifs are shown within boxes and the delimitations of the three domains are indicated with arrows. Asterisks, colons, and periods indicate identical, conserved, and semiconserved residues, respectively. (0.06 MB PDF) [file ppat.1000353.s001.pdf]

Table S1. Characterization of *in vitro* and *in vivo* ceftazidime resistant mutants**.**

Strain	Description[a]	CAZ MIC (μg/ml)	Comp. pUCPAD/ pUCPADE[b]	*ampD* mutations [c] [effect]	*ampR* mutations [effect]	*dacB* mutations [effect]
**PAO1**	Reference strain	1	-/-	None	None	None
**PAOΔ*mutS***	PAO1 *mutS* knockout mutant	1	-/-	None	None	None
**1A1**	PAO1-*in vitro*-CAZ4	32	-/+	None	None	G-A nt 819 [W273X]
**1A2**	PAO1-*in vitro*-CAZ4	24	-/+	None	None	G-T nt 1102 [G368X]
**1A3**	PAO1-*in vitro*-CAZ4	32	-/+	None	None	Ins 1 bp (C) nt 336 [frameshift]
**1A4**	PAO1-*in vitro*-CAZ4	24	-/+	None	None	A-G nt 965 [Q322R]
**1A5**	PAO1-*in vitro*-CAZ4	24	-/+	None	None	Del. nts 1149-1231 [Deletion/frameshift]
**1D4**	PAO1-*in vitro*-CAZ16	32	-/+	None	None	A-C nt 235 [T79P]
**1D5**	PAO1-*in vitro*-CAZ16	32	-/+	None	None	Del. nts 592-593 [frameshift]
**1D6**	PAO1-*in vitro*-CAZ16	32	-/+	None	None	Del. nts 983-993 [deletion/frameshift]
**1D7**	PAO1-*in vitro*-CAZ16	32	-/+	None	None	Del. nts 1069-1138 [Del. T357-E379]
**1D8**	PAO1-*in vitro*-CAZ16	32	-/+	None	None	Duplication nts 800-856 [Insertion]
**1B2**	PAOΔ*mutS* -*in vitro*-CAZ4	96	-/+	None	None	C-T nt 109 [Q37X]
**1B3**	PAOΔ*mutS* -*in vitro*-CAZ4	32	-/+	None	None	C-T nt 215 [S72L]

**1B4**	PAOΔ*mutS* -*in vitro*-CAZ4	24	-/+	None	None	C-T nt 1318 [R440C]
**1B5**	PAOΔ*mutS* -*in vitro*-CAZ4	32	-/+	None	None	G-A nt 1310 [G437D]
**1B6**	PAOΔ*mutS* -*in vitro*-CAZ4	64	-/+	None	None	T-C nt 971 [F324S]
**1C3**	PAOΔ*mutS* -*in vitro*-CAZ16	64	-/+	None	None	T-C nt 1340 [W447R]
**1C4**	PAOΔ*mutS* -*in vitro*-CAZ16	48	-/+	None	None	T-C nt 911 [L304P]
**1C5**	PAOΔ*mutS* -*in vitro*-CAZ16	64	-/+	None	None	T-C nt 611 [L204P]
**1C6**	PAOΔ*mutS* -*in vitro*-CAZ16	64	-/+	None	None	G-A nt 1310 [G437D]
**1C7**	PAOΔ*mutS* -*in vitro*-CAZ16	64	-/+	None	None	C-T nt 1109 [S370L]
**2A2**	PAO1-*in vivo*-CAZ4	48	-/+	None	None	Del nts 1074-1078 [Deletion/frameshift]
**2A4**	PAO1-*in vivo*-CAZ4	48	-/+	None	None	G-A nt 1339 [W459X]
**2D3**	PAO1-*in vivo*-CAZ4	48	-/+	None	None	A-C nt 1282 [T428P]
**2D4**	PAO1-*in vivo*-CAZ4	32	-/+	None	None	None
**2D5**	PAO1-*in vivo*-CAZ4	48	-/+	None	None	Del nts 1351-1385 [Del. A451-S461]
**2A3**	PAO1-*in vivo*-CAZ16	24	-/+	None	None	Del nts 342-792 [Del K114-Y264]
**2A7**	PAOΔ*mutS* -*in vivo*-CAZ4	48	+/+	C-T nt 130 [Q44X]	None	None
**2A9**	PAOΔ*mutS* -*in vivo*-CAZ4	64	+/+	T-C nt -23 [mutation in promoter (-10 box)]	None	None
**2B2**	PAOΔ*mutS* -*in vivo*-CAZ4	64	+/+	C-T nt 503 [P168L]	None	None

**2C7**	PAOΔ*mutS* -*in vivo*-CAZ4	96	-/+	None	None	G-A nt 1050 [W350X]
**2C8**	PAOΔ*mutS* -*in vivo*-CAZ4	32	+/+	C-T nt 341 [S114F]	None	None
**2A8**	PAOΔ*mutS* -*in vivo*-CAZ16	96	-/+	None	None	T-G nt 978 [N316X]
**2B1**	PAOΔ*mutS* -*in vivo*-CAZ16	96	-/+	None	None	A-G nt 224 [K75R]
**2B3**	PAOΔ*mutS* -*in vivo*-CAZ16	96	-/+	None	None	G-A nt 1164 [W388X]
**2C9**	PAOΔ*mutS* -*in vivo*-CAZ16	64	-/+	None	None	G-A nt 1050 [W350X]
**2D1**	PAOΔ*mutS* -*in vivo*-CAZ16	96	-/+	None	None	None

a The mutants were obtained (Plasencia *et al* 2007), *in vitro* (one-step spontaneous mutants) or *in vivo* (after 3 days of treatment with humanized ceftazidime regimen in mouse model of lung infection), at two ceftazidime (CAZ) concentrations (4 and 16 μg/ml), and from the wild-type strain PAO1 (normal mutation rate supply) or its *mutS* deficient derivative PAOΔ*mutS* (high mutation rate supply).

[b] For complementation experiments, plasmids pUCPAD (harbouring the wild-type *ampD* gene), pUCPADE (harbouring the complete wild-type *ampDE* operon) or pUCP24 (cloning vector) were electroporated into the different β-lactam resistant strains or PAO1 (as control). Complementation was considered positive when the MICs of ceftazidime for the transformants were at least 3 two-fold dilutions lower than those of the parent strains. Additionally, plasmids pUCPADE2A1, pUCPADE1C5 or pUCPADE2C2 containing a non functional *ampD* and a wild-type *ampE* were electroporated in selected mutants, failing in all cases to complement the resistance phenotype.

[c] Mutations or a modified expression of *ampE*, *ampDh2*, or *ampDh3* was not detected in any of the mutants.

References

Plasencia V, Borrell N, Maciá MD, Moya B, Pérez JL, et al. (2007) Influence of high mutation rates on the mechanisms and dynamics of in vitro and in vivo resistance development to single or combined antipseudomonal agents. Antimicrob Agents Chemother 51: 2574-2581.
