# Supplementary material for: β-Lactam Resistance Response Triggered by Inactivation of a Nonessential Penicillin-Binding Protein
Source: PLoS Pathog. 2009 Mar 27;5(3):e1000353. doi: 10.1371/journal.ppat.1000353 (PMC2654508; doi:10.1371/journal.ppat.1000353)
Supplement: Table S2 — Strains and plasmids used or constructed in this study (0.06 MB PDF) [file ppat.1000353.s002.pdf]

Table S2. Strains and plasmids used or constructed in this study.

Strain or plasmid	Genotype/relevant characteristics	Reference or source
P. aeruginosa		
PAO1	Reference strain completely sequenced	Stover *et al* (2000)
PAOΔmutS	PAO1 Δ*mutS*::*km*<br>MutS is a component of the DNA mismatch repair system. Its inactivation increases spontaneous mutation rate by 2-3 log	Oliver *et al* (2004)
PAΔdacB	PAO1 Δ*dacB*::*lox*<br>*dacB* encodes the nonessential Penicillin Binding Protein 4	This work
PAΔD	PAO1 Δ*ampD*::*lox*<br>AmpD is a N-acetyl-anhydromuramyl-L-alanine amidase involved in peptidoglycan recycling. It is a negative regulator of AmpC expression	Juan *et al* (2006)
PAΔE	PAO1 Δ*ampE*::*lox*<br>*ampE* forms the bicistronic *ampDE* operon together with *ampD*. AmpE is an inner membrane-bound sensory transducer that modulates AmpD activity	This Work
PAΔDE	PAO1 Δ*ampD*::*lox* Δ*ampE*::*lox*	This Work
PAΔR	PAO1 Δ*ampR*::*lox*<br>AmpR is LysR-type transcriptional regulator required for *ampC* induction	This work
PAΔC	PAO1 Δ*ampC*::*lox*<br>*ampC* encodes the chromosomal β-lactamase AmpC	Moya *et al* (2008)
PAΔcreBC	PAO1 Δ*creBC*::*lox*<br>CreBC is a two-component response regulator	This Work
PAΔcreD	PAO1 Δ*creD*::*lox*<br>Inner membrane protein of unknown function known to be regulated by the CreBC system	This Work
PAΔDDh2Dh3	PAO1 Δ*ampD*::*lox* Δ*ampDh2*::*lox* Δ*ampDh3*::*lox*<br>AmpDh2 and AmpDh3 are the two additional AmpD homologues of *P. aeruginosa*	Juan *et al* (2006)
PAΔDDh2Dh3R	PAO1 Δ*ampD*::*lox* Δ*ampDh2*::*lox* Δ*ampDh3*::*lox* Δ*ampR*::*lox*	This Work

PAΔDDh2Dh3C	PAO1 Δ*ampD*::*lox* Δ*ampDh2*::*lox* Δ*ampDh3*::*lox* Δ*ampC*::*lox*	Moya *et al* (2008)
PAΔDDh2Dh3creBC	PAO1 Δ*ampD*::*lox* Δ*ampDh2*::*lox* Δ*ampDh3*::*lox* Δ*creBC*::*lox*	This Work
1A1	PAO1 one-step spontaneous CAZ resistant mutant obtained *in vitro*	Plasencia *et al* (2007)
1A1ΔD	1A1 Δ*ampD*::*lox*	This Work
1A1ΔE	1A1 Δ*ampE*::*lox*	This Work
1A1ΔR	1A1 Δ*ampR*::*lox*	This Work
1A1ΔcreBC	1A1 Δ*creBC*::*lox*	This Work
1A1ΔC	1A1 Δ*ampC*::*lox*	This Work
2A2	PAO1 CAZ resistant mutant obtained *in vivo* (mouse model of lung infection)	Plasencia *et al* (2007)
2A2ΔcreBC	2A2 Δ*creBC*::*lox*	This Work
2A2ΔC	2A2 Δ*ampC*::*lox*	This Work
E. coli		
XL-1 blue	F'::Tn10 *proA*$^+$*B*$^+$ *lac1*^{q} Δ*(lacZ)M15/recA1 endA1 gyrA96* (Nal^R) *thi hsdR17* ($r_k^-m_k^-$) *mcrB1*	Laboratory collection
S17.1	RecA pro (RP4-2Tet:: Mu Kan::Tn7)	Laboratory collection
**Plasmids**		
pUCP24	Gm^R, pUC18-based *Escherichia-Pseudomonas* shuttle vector	West *et al* (1994)
pUCPAD	Gm^R, pUCP24 containing wild-type *ampD* from PAO1	Juan *et al* (2005)
pUCPADE	Gm^R, pUCP24 containing the complete wild-type *ampDE* operon from PAO1	Juan *et al* (2005)
pUCPADE2A1	Gm^R, pUCP24 containing the complete *ampDE* operon from *P. aeruginosa* clinical isolate 2A1. *ampD* gene from this strain is nonfunctional due to a frameshift mutation [1-bp insertion (C) in nt 481]. *ampE* shows wild-type sequence. Reported in Juan *et al* (2005)	This Work
pUCPADE2C2	Gm^R, pUCP24 containing the complete *ampDE* operon from *P. aeruginosa* clinical isolate 2C2. *ampD* gene from this strain is nonfunctional due to a frameshift mutation [1-bp deletion (C) in nt 449]. *ampE* shows wild-type sequence. Reported in Juan *et al* (2005)	This Work
pUCPADE1C5	Gm^R, pUCP24 containing the complete *ampDE* operon from *P. aeruginosa* clinical isolate 1C5. *ampD* gene from this strain is nonfunctional due to a nonsense mutation (C-T mutation in nt 463). *ampE* shows wild-type sequence. Reported in Juan *et al* (2005)	This Work
pUCPdB	Gm^R, pUCP24 containing wild-type *dacB* from PAO1	This work
pEX100Tlink	Ap^R, *sacB*, pUC19-based gene replacement vector with a MCS	Quénée *et al* (2005)
pUCGm*lox*	Ap^R, Gm^R, pUC18-based vector containing the *lox* flanked *aacC1* gene	Quénée *et al* (2005)

pCM157	Tc^R, *cre* expression vector	Quénée *et al* (2005)
pEXADGm	pEX100Tlink containing 5'and 3' flanking sequence of *ampD* ::Gm*lox*	Juan *et al* (2006)
pEXdacBGm	pEX100Tlink containing 5'and 3' flanking sequence of *dacB* ::Gm*lox*	This work
pEXcreBCGm	pEX100Tlink containing 5'and 3' flanking sequence of *creBC* ::Gm*lox*	This work
pEXcreDGm	pEX100Tlink containing 5'and 3' flanking sequence of *creD* ::Gm*lox*	This work
pEXARGm	pEX100Tlink containing 5'and 3' flanking sequence of *ampR* ::Gm*lox*	This work
pEXACGm	pEX100Tlink containing 5'and 3' flanking sequence of *ampC* ::Gm*lox*	Moya *et al* (2008)
pEXAEGm	pEX100Tlink containing 5'and 3' flanking sequence of *ampE* ::Gm*lox*	This work

References

Juan C, Macia MD, Gutierrez O, Vidal C, Perez JL,et al. (2005) Molecular mechanisms of β-lactam resistance mediated by AmpC hyperproduction in *Pseudomonas aeruginosa* clinical strains. Antimicrob Agents Chemother 49: 4733-4738.

Juan C, Moya B, Perez JL, Oliver A (2006) Stepwise upregulation of the *Pseudomonas aeruginosa* chromosomal cephalosporinase conferring high level beta-lactam resistance involves three AmpD homologues. Antimicrob Agents Chemother 50: 1780-1787.

Moya B, Juan C, Alberti S, Perez JL, Oliver A (2008) Benefit of having multiple *ampD* genes for acquiring β-lactam resistance without losing fitness and virulence in *Pseudomonas aeruginosa*. Antimicrob Agents Chemother 52: 3694-3700.

Oliver A, Levin BR, Juan C, Baquero F, Blázquez J (2004) Hypermutation and the preexistence of antibiotic-resistant *Pseudomonas aeruginosa* mutants: implications for susceptibility testing and treatment of chronic infections. Antimicrob Agents Chemother 48: 4226-4233**.**

Plasencia V, Borrell N, Maciá MD, Moya B, Pérez JL, et al. (2007) Influence of high mutation rates on the mechanisms and dynamics of in vitro and in vivo resistance development to single or combined antipseudomonal agents. Antimicrob Agents Chemother 51: 2574-2581.

Quénée L, LamotteD, Polack B (2005) Combined *sacB*-based negative selection and cre-lox antibiotic marker recycling for efficient gene deletion in *Pseudomonas aeruginosa.* BioTechniques 38: 63-67.

Stover CK, Pham XQ, Erwin AL, Mizoguchi SD, Warrener P, et al. (2000) Complete genome sequence of *Pseudomonas aeruginosa* PAO1: an opportunistic pathogen. Nature 406: 959-964.

West SE, Schweizer HP, Dall C, Sample AK, Runyen-Janecky LJ (1994) Construction of improved *Escherichia-Pseudomonas* shuttle vectors derived from pUC18/19 and sequence of the region required for their replication in *Pseudomonas aeruginosa*. Gene 148: 81-86.
