# Supplementary material for: β-Lactam Resistance Response Triggered by Inactivation of a Nonessential Penicillin-Binding Protein
Source: PLoS Pathog. 2009 Mar 27;5(3):e1000353. doi: 10.1371/journal.ppat.1000353 (PMC2654508; doi:10.1371/journal.ppat.1000353)
Supplement: Table S3 — Primers used in this work (0.04 MB PDF) [file ppat.1000353.s003.pdf]

Table S3. Primers used in this work

Primer	Sequence (5`-3`)[a]	Location[b]	PCR product size (bp)	Use	Reference or source
ACrnaF<br>ACrnaR	GGGCTGGCCTCGAAAGAGGAC<br>GCACCGAGTCGGGGAACTGCA	208 to 228<br>434 to 454	246	Quantification of *ampC* mRNA	Juan *et al* (2006)
ADrnaF<br>ADrnaR	CGCGCATTTCCTCATCGAACGC<br>TCGCAGTGGCCCTGGATGCG	225 to 246<br>457 to 476	251	Quantification of *ampD* mRNA	Juan *et al* (2006)
AErnaF<br>AErnaR	CCGCTGCACCTTCTGGTGGTG<br>GCCACCGGGCCGAGCAAGGCA	245 to 265<br>488 to 508	246	Quantification of *ampE* mRNA	This work
creDrnaF<br>creDrnaR	CGGCGTGCTGCAGGATATCGC<br>TGTCGACGTGGTACAGGCGCG	114 to 134<br>344 to 364	251	Quantification of *creD* mRNA	This work
ADF<br>ADR	GTACGCCTGCTGGACGATG<br>GAGGGCAGATCCTCGACCAG	-233 to -253<br>707 to 726	916	*ampD* amplification and sequencing	Juan *et al* (2005)
ADH2F<br>ADH2R	GGGCAGCGCGGTCAGGC<br>TCAGGAAGTCGGCACCGCC	-19 to -39<br>762 to 780	815	*ampDh2* amplification and sequencing	Juan *et al* (2006)
ADH3F<br>ADH3R	TTGGCCGGCCCCTGAAC<br>GCGACGACCTGAGCGACG	-81 to -97<br>845 to 862	960	*ampDh3* amplification and sequencing	Juan *et al* (2006)
AEF<br>AER	GCCTGGACCCGAACGAAC<br>TCAGAGGAACAGCGCGCAG	-93 to -111<br>1013 to 1032	1231	*ampE* amplification and sequencing	This work
ARF	GTCGACCCAGTGCCTTCAGG	1138 to 1156	1391	*ampR* and *amp*C-*ampR* intergenic region amplification and sequencing	Juan *et al* (2005)

ARR	CTCGAGAGCGAGATCGTTGC	-239 to -220			
CreBF	CACCAAGGGCTTGCGCA	-147 to -166	1066	*creB* amplification and sequencing	This work
CreBR	TCGAGCATCTCCGGCAGG	887 to 903			
CreBIR	CTGGAACGGCCCGCTC	393 to 408		*creB* sequencing	This work
CreCF	CAGCCCGGACCACGCCTG	-18 to -1	1560	*creC* amplification and sequencing	This work
CreCR	CCAGCGTGCGGTTCATG	1526 to 1542			
CreCIF	CCTGCCGGAGATGCTCG	198 to 214		*creC* sequencing	This Work
CreDF	GGCTGCCGCGCGGCTGA	-118 to -102	1477	*creD* amplification and sequencing	This work
CreDR	TCAGGCCCTGGCGGGTAC	1342 to 1359			
CreDIF2	GTCGACAACAAGGTTGCCGG	358 to 377		*creD* sequencing	This work
CreDIR	GATGCCATAGTGCGCAGGC	389 to 408			
CreDIF	GCGACGGCTTCCAGGCGC	842 to 859			
DACB-F	CGACCATTCGGCGATATGAC	-178 to -159	1721	*dacB* amplification and sequencing	This work
DACB-R	CGCGTAATCCGAAGATCCATC	1526 to 1546			
DACB-I-R	GTCGCGCATCAGCAGCCAG	378 to 396		*dacB* sequencing	This work
DACB-I-F	GCCAGGGCAGCGTACCGC	854 to 871			
DACB-I-F2	GTGCTCAACGGCAACCTCTAC	316 to 336			
AD-F0ERI	TCGAATTCGTCCTGACTTCGCCGGAC	-478 to -496	523	AmpD inactivation	Juan *et al* (2006)
AD-R3HDIII	TCAAGCTTCACGGACCCAGCCGGTAAC	16 to 34			
AD-F2HDIII	TCAAGCTTGCCTGGACCCGAACGAAC	170 to 187	512		
AD-RBHI	TCGGATCCGAGGGCAGATCCTCGACCAG	663 to 682			
DACB-F-ERI	TCGAATTCCGACCATTCGGCGATATGAC	-175 to -156	571	DacB inactivation	This work
DACB-I-R-HD3	TCAAGCTTGTCGCGCATCAGCAGCCAG	378 to 396			
DACB-I-F-HD3	TCAAGCTTGCCAGGGCAGCGTACCGC	854 to 871	693		
DACB-R-BHI	TCGGATCCCGCGTAATCCGAAGATCCATC	1526 to 1546			
AmpC-F-ERI	TCGAATTCGCGCGCAGGGCGTTCAG	-186 to -169	415	AmpC inactivation	Moya *et al* (2008)
AmpC-I-R-HDIII	TCAAGCTTCGTCCTCTTACGAGGCCAG	210 to 229			
AmpC-I-F-HDIII	TCAAGCTTCAGGGCAGCCGCTTCGAC	366 to 384	432		
AmpC-R-BHI	TCGGATCCCAGGTTGGCATCGACGAAG	779 to 798			
AEXBI-F1	GCTCTAGACGCATCCAGGGCCACTGCG	-93 to -111	488	AmpE inactivation	This work
AEHDIII-R1	TCAAGCTTCGTTCGGCGACATGGAAGGC	358 to 377			
AEHDIII-F2	TCAAGCTTGCACGGCCATCTGCTCTGGC	430 to 449	606		
AEERI-R2	TCGAATTCTTGACGCACGGAGTCCGCTC	1016 to 1035			

AmpR-F-ERI	TCGAATTCCACCAGGTGAAGAGCCTCG	123 to 141	424	AmpR inactivation	This work
AmpR-I-R-HDIII	TCAAGCTTGACTGTGCAACTGGGCGG	552 to 572			
AmpR-I-F-HDIII	TCAAGCTTCAGGGTGTCGGCGTGGC	728 to 744	409		
AmpR-R-BHI	TCGGATCCGCCTATGCCGCCAGCCTG	1140 to 1157			
CreB-F-ERI	TCGAATTCCACCAAGGGCTTGCGCA	-147 to -166	571	CreBC inactivation	This work
CreB-I-R-HD3	TCAAGCTTCTGGAACGGCCCGCTC	393 to 408			
CreC-I-F-HD3	TCAAGCTTCCTGCCGGAGATGCTCG	198 to 214	466		
CreC-Rb-BHI	TCGGATCCGCTGACCGCCTGGGCG	1337 to 1352			
creD-F-BHI	TCGGATCCGGCTGCCGCGCGGCTGA	-118 to -102	526	CreD inactivation	This work
creD-I-R-HD3	TCAAGCTTGATGCCATAGTGCGCAGGC	390 to 408			
creD-I-F-HD3	TCAAGCTTGATCACCGCCCAGGGCTTC	747 to 765	613		
creD-R-ERI	TCGAATTCTCAGGCCCTGGCGGGTAC	1342 to 1359			

[a] Sites for restriction endonucleases are underlined.

[b] Location of the primers respect to the start codon of the corresponding genes.

References

Juan C, Macia MD, Gutierrez O, Vidal C, Perez JL, et al. (2005) Molecular mechanisms of β-lactam resistance mediated by AmpC hyperproduction in *Pseudomonas aeruginosa* clinical strains. Antimicrob Agents Chemother 49: 4733-4738.

Juan C, Moya B, Perez JL, Oliver A (2006) Stepwise upregulation of the *Pseudomonas aeruginosa* chromosomal cephalosporinase conferring high level beta-lactam resistance involves three AmpD homologues. Antimicrob Agents Chemother 50: 1780-1787.

Moya B, Juan C, Alberti S, Perez JL, Oliver A (2008) Benefit of having multiple *ampD* genes for acquiring β-lactam resistance without losing fitness and virulence in *Pseudomonas aeruginosa*. Antimicrob Agents Chemother 52: 3694-3700.
