# Supplementary material for: β-Lactam Resistance Response Triggered by Inactivation of a Nonessential Penicillin-Binding Protein
Source: PLoS Pathog. 2009 Mar 27;5(3):e1000353. doi: 10.1371/journal.ppat.1000353 (PMC2654508; doi:10.1371/journal.ppat.1000353)
Supplement: Figure S1 — Clustal W 2.0.8 multiple-sequence alignment of DacB from E. coli K12 and P. aeruginosa PAO1 (0.03 MB PDF) [file ppat.1000353.s004.pdf]

```
                                        Domain I
                                     ------------->
E. coli           ---------MRFSRFIIGLTSCIAFSVQAANVDEYI-TQLPAGANLALMVQKVGASAPAI 50
P.aeruginosa      MFKSLRTLAFATLLPFALPTLAQVNATLPANVQKALQTNKLTGNDLSLVLIPLDGPGNPT 60
                           :     :   *   . :: .*** .   *:  :* :*:*::  :.... .

                                        Domain II
                                      ------------->
E.coli            DYHSQQMALPASTQKVITALAALIQLGPDFRFTTTLETKGNVENGVLKGDLVARFGADPT 110
P.aeruginosa      YYNADVSVNPASTMKLFTTYAALEMLGPTYQWKTEFYTDGQLKNGVLNGNLYLKGGGDPK 120
                   *:::  . **** *::*: ***  *** :::.* : *.*:::****:*:*  : *.**.

E. coli           LKRQDIRNMVATLKKSGVNQIDGNVLIDTSIFASHDKAPGWPWN--DMTQCFSAPPAAAI 168
P. aeruginosa     LNMEKLWLLMRDLRANGVTKVTGDLVLDRSYFN-IPQLPVFNDDGGDDTKPFLVGPDSLL 179
                  *: :.:  ::  *: .**.:: *::::* * *    : * :  :  * *: * . * : :


                   Domain III
                  ---------->
E. coli           VDRNCFSVSLYSAPKPGDMAFIRVASYYPVTMFSQVRTLPRGSAEAQYCELDVVPGDLNR 228
P. aeruginosa     VNLKSVRMVVRTDGNKVNVQMDPPLANVRIDNQVKMTAPATCPAWPKLRFSPVTQFDGTT 239
                  *: :.. : : :  :  :: :    :   :    :: : .  .* .:     *.  * .


                      Domain III
                     <----------
E. coli           FTLTGCLPQRSEPLPLAFAVQDGASYAGAILKDELKQAGITWSGTLLRQTQVNEPGTVVA 288
P. aeruginosa     LLATGQIPQGCS-AQTYMSLLDHPGYTAGAVRGIWQELGGSILG-KDRQGSVPRNATLIA 297
                  :  ** :** ..     ::: * ..*:.. ::.  :: * :  *   ** .* . .*::*

                   Domain II
                  <-----
E. coli           SKQSAPLHDLLKIMLKKSDNMIADTVFRMIG-HARFNVPGTWRAGSDAVRQILRQQAGVD 347
P. aeruginosa     KAFSPDLVEIIRDINKYSNNTMARQLFLSIGAQFRNSADGDDAQAAQRVVRQWLARKGIT 357
                  .  *. * :::: : * *:* :*  :*  ** : * .. *    .:: * :    : *:

E. coli           IGNTIIADGSGLSRHNLIAPATMMQVLQYIAQHDNELNFISMLPLAGYDGSLQYRAGLHQ 407
P. aeruginosa     APRLVMENGSGLSRQERVSAREMAAMLQAAWHSPYAAEYISSLPLAGLDGTMRKR--LRR 415
                    . :: :******:: ::.  *  :**   :     ::** ***** **::: *  *::

E.coli            AGVDGKVSAKTGSLQGVYNLAGFITTASGQRMAFVQYLSGYAVEPADQRNRRIPLVRFES 467
P. aeruginosa     TALVGEAHVKTGTLNTVRALAGFSRDASGHNWVVVAILNSPRPWGASAILDQVLLSLHAR 475
                  :.: *:. .***:*: *  ****   ***:. ..*  *..     *.    :: *  .

                    Domain I
                  <---------
E. coli           RLYKDIYQNN 477
P. aeruginosa     K--------- 476
                  :
```

Figure S1. Clustal W 2.0.8 multiple sequence alignment of DacB from *E. coli* K12 and *P. aeruginosa* PAO1. The sequences corresponding to the signal peptides are shown in italics. SXXK, SXN, and KTG motifs are shown within boxes and the delimitations of the three domains are indicated with arrows. Asteriks, colons, and periods indicate identical, conserved, and semiconserved residues, respectively.
